# Perceived Neighborhood Environmental Factors Related to Sarcopenia in Urban-Dwelling Older Adults

**DOI:** 10.1093/geroni/igaa057.1418

**Published:** 2020-12-16

**Authors:** Yuri Seo, Miji Kim, Hayoung Shim, Heeeun Jung, Seoyoon Jane Lee, Chang Won Won

**Affiliations:** 1 Graduate School, Kyung Hee University, Seoul, Republic of Korea; 2 College of Medicine, Kyung Hee University, Seoul, Republic of Korea; 3 Kyung Hee University, Seoul, Korea, Seoul, Republic of Korea; 4 Elderly Frailty Research Center, Kyung Hee University, Seoul, Republic of Korea; 5 Kyung Hee University Medical Center, Seoul, Korea, Seoul, Republic of Korea

## Abstract

Sarcopenia is common among older individuals and has adverse health outcomes. However, little is known about its association with neighborhood environmental factors. We explored the relationship between sarcopenia and neighborhood environmental factors among community-dwelling older adults aged 70–84 years in urban areas in the Korean Frailty and Aging Cohort Study. There were 1,776 participants in this cross-sectional study (mean age 75.9±3.8 years, 54.1% women). Sarcopenia was defined using the Asian Working Group for Sarcopenia guidelines. The neighborhood environmental factors were assessed using the 17-item Environmental Module of the International Physical Activity Questionnaire (IPAQ-E). The prevalence of sarcopenia was 22.5%. In the multivariate analysis adjusted for potential confounders, compared to the 5th quintile of the IPAQ-E score, the odds ratio (OR and 95% confidence interval [CI]) for sarcopenia in the 1st, 2nd, 3rd and 4th quintile were 2.14 (1.41-3.26), 1.70 (1.11-2.61), 1.76 (1.16-2.68) and 1.62 (1.07-2.47), respectively. Sarcopenia was associated with environmental factors including access to destinations (β = -0.015) and neighborhood safety (β = -0.008) (all p<0.05). Furthermore, no access to public transportation (OR 2.05, 95% CI 1.20-3.50), poor access to recreational facilities (OR 1.40, 95% CI 1.02-1.92), no presence of destination (OR 1.53, 95% CI 1.07-2.21), hill hazard (OR 1.34, 95% CI 1.02-1.77), and lack of safety from traffic (OR 1.35, 95% CI 1.02-1.79) was associated with an increased risk of sarcopenia. Our study suggests that neighborhood environmental characteristics are associated with sarcopenia and better neighborhood environmental strategies can help prevent sarcopenia among older adults.

